# A New Approach for Construction of Geodemographic Segmentation Model and Prediction Analysis

**DOI:** 10.1155/2019/9252837

**Published:** 2019-05-13

**Authors:** Hoang Viet Long, Le Hoang Son, Manju Khari, Kanika Arora, Siddharth Chopra, Raghvendra Kumar, Tuong Le, Sung Wook Baik

**Affiliations:** ^1^Division of Computational Mathematics and Engineering, Institute for Computational Science, Ton Duc Thang University, Ho Chi Minh City, Vietnam; ^2^Faculty of Mathematics and Statistics, Ton Duc Thang University, Ho Chi Minh City, Vietnam; ^3^VNU Information Technology Institute, Vietnam National University, Hanoi, Vietnam; ^4^Department of Computer Science and Engineering, AIACT&R, New Delhi, India; ^5^Computer Science and Engineering Department, LNCT College, Bhopal, MP, India; ^6^Digital Contents Research Institute, Sejong University, Seoul 143-747, Republic of Korea

## Abstract

Customer retention is invariably the top priority of all consumer businesses, and certainly it is one of the most critical challenges as well. Identifying and gaining insights into the most probable cause of churn can save from five to ten times in terms of cost for the company compared with finding new customers. Therefore, this study introduces a full-fledged geodemographic segmentation model, assessing it, testing it, and deriving insights from it. A bank dataset consisting 11,000 instances, which consists of 10,000 instances for training and 10,000 instances for testing, with 14 attributes, has been used, and the likelihood of a person staying with the bank or leaving the bank is computed with the help of logistic regression. Base on the proposed model, insights are drawn and recommendations are provided. Stepwise logistic regression methods, namely, backward elimination method, forward selection method, and bidirectional model are constructed and contrasted to choose the best among them. Future forecasting of the models has been done by using cumulative accuracy profile (CAP) curve analysis.

## 1. Introduction

Churn is the challenging problem that every enterprise sector as well as consumer businesses have to deal with. A disproportionate amount of customer churn could drive a company to change its policy decisions. Therefore, retention of customers is a priority for almost all organizations as gaining new customers can be much more expensive than keeping the existing ones. There are many data analysis methods that have been proposed to find the valuable knowledge from the data including data mining approaches in [1–5] and machine learning approaches in [6–10]. In machine learning, geodemographic segmentation [11–14] is an interesting topic with many applications. As a solution, the company seeks to predict the high-incline churners and offers them discounts, offers, or facilities to address their special requirements. This requires them to gain power insights from the data about the potential reasons for the churn that can help estimate churn risk and then avoid or minimize it in the future. Thus, a thoroughly constructed churn model based on the geological, demographic, or behavior information taken by the customers can drive the company's decision making towards higher profits and improvement areas. The enterprises also have to take into account the level and cost of the intervention, risk (and associated risk tolerance), and plausible customer segmentation.

The process of clustering different individuals within a population into groups based on their geographical and analytical information is what constitutes a geodemographic segmentation model [15]. The company needs this information to fully understand its customer's behaviors that might predict the factors leading to such an unusual and excessive churn. The consumer data can contain a huge number of predictors or input variables that might not all be significant in analyzing the churn rate. Regression techniques can help analysts or market researchers to eliminate irrelevant variables and figure out the best technique to build predictive models. Once the regression equation is formulated, its predictive ability can be examined using goodness of fit criterion. The feature selection process via stepwise logistic regression minimizes dimensionality and effectively reduces costs and data volume.

Identifying customer churn factors including customer complaints, core service failures, service usage, and loyalty programs may be useful in improving company operations and policies in terms of their marketing strategies as well as customer churn prevention programs [16]. A sophisticated and mature industry would opt to recognize every customer with the likelihood of leaving the bank and address the top *N* ones. CAP curve analysis helps in determining these top *N* customers who are at high level of leaving. It is also used to handle model deterioration over time, determining accuracy and check for overfitting of the data.

This paper is an attempt to solve the churn rate problem for user dataset for a bank collected over a period of six months. Stepwise logistic regression technique is first used to select the most significant variables among all the variables in the dataset. This is done by using three techniques, forward selection approach, backward elimination approach, and bidirectional approach. These techniques are then compared according to their accuracy, overfitting of the data, and forecasting ability through CAP curve analysis. Predictive analysis is then performed by using various classification techniques.

The rest of this study is structured as follows.  Section 2 presents some related works including geodemographic segmentation model, stepwise regression methods for feature selection, various selection fit criteria for checking the goodness of the model, confusion matrices, multicollinearity, and future forecasting using CAP curve analysis.  Section 3 introduces the dataset and its variables, the techniques to be used for feature selection, and the tools used for that purpose.  Section 4 describes the observations such as contrast among the models, accuracies of classification techniques, effect of feature selection, and insights gained from the model along with visualization graphs.  Section 5 offers insights taken from the model construction and prediction.  Section 6 gives the conclusion of this research and future works.

## 2. Method Descriptions

### 2.1. Geodemographic Segmentation Model

In marketing and computer science, geodemographic segmentation is a multivariate statistical classification technique for discovering whether the individuals of a population fall into different groups by making quantitative comparisons of multiple characteristics with the assumption that the differences within any group should be less than the differences between groups [4, 11, 15]. It means that audience preference is taken into the account based on their geographic location like which country or area they live in and all their demographic information such as their income, gender, marital status, and their tenure with the company. Such information based on some similarities of these customers can help to segment them in groups to predict the future.

### 2.2. Stepwise Regression Approach

In statistical modelling, regression analysis is a set of statistical processes for estimating the relationship between a dependent predictor and one or more independent predictors (or “variables”). Regression analysis helps to understand how the value of the dependent predictor (or “criterion variable”) varies when any one of the independent variables changes and while the other independent variables remain constant. Regression techniques help us to find out which predictors affect the target value, and which do not. It indicates relationships between the independent predictor and dependent predictor and the strength of impact of multiple independent variables on a dependent variable [17].

### 2.3. Selection of Fit Criteria

The selection criterion is used to analyze the goodness of the constructing model by stopping the stepwise regression analysis at a definite time. It is also used to determine when a new independent variable needs to be added and an already selected variable needs to be deleted from the regression model. Some common criteria are AIC (Akaike information criterion) [18], BIC (Bayesian information criterion) [19], *R*^2^ (coefficient of determination), and adjusted *R* [18].

### 2.4. Confusion Matrix

A confusion matrix or an error matrix is a table layout that allows visualization of the performance of a classification algorithm. Calculating a confusion matrix can provide a better idea about the classification model and the type of errors in it. The basic terms associated with a confusion matrix are as follows: (1) true positives: these are cases which were predicted to occur by the model, and they actually occurred. (2) true negatives: these are cases which were predicted not to occur, and they did not occur. (3) False positives: also known as Type I error, these are the cases which were predicted to occur by the model, but they did not. (4) False negatives: also known as Type II error, these are the cases which were predicted not to occur, but they actually did. A confusion matrix for 10,000 customers is shown in Table 1.

A confusion matrix is used to compute accuracy of the classifier (how often the model was right), misclassification rate (how often the model was wrong), true positive rate (how often does it predict true positives), false positive rate (how often does it predict false positives), specificity (how often does it predict true negatives), precision (how often does it predict true positives), and prevalence (how often does the occurrence condition actually occur in the sample).

### 2.5. Multicollinearity

Multicollinearity is a statistical phenomenon in which there exists a relationship between the independent variables which makes it difficult to come up with reliable estimates of their individual coefficients. It inflates the variances of the parameter estimates, and hence this may lead to lack of statistical significance of individual predictor variables even though the overall model may be significant. It might result in incorrect conclusions causing the model to break. The multicollinearity in statistical modelling can be detected in three ways:

- *Examination of Correlation Matrix*. A correlation matrix investigates the interdependency between all multiple independent predictors at the same time. Correlation can be between −1 to unity, and it is basically a parameter that tells that if one predictor changes, how much will the other predictor change, as well and higher this parameter is, the more is the indication of collinearity. A typical correlation matrix with four predictors is shown in Table 2.
- *Variance Inflation Factors (VIF)*. The variance inflation factor (VIF) quantifies the severity of multicollinearity in an ordinary least-squares regression analysis. Practically, if the value of VIF exceeds 5 or 10, it implies that the associated regression coefficients are poorly estimated because of multicollinearity. Values greater than 10.0 indicate multicollinearity, as shown in Table 3.
- *Eigen system Analysis of Correlation Matrix*. If one or more of the eigenvalues are small (close to zero) and the corresponding condition number is large, then it indicates multicollinearity.

### 2.6. Cumulative Accuracy Profile

**Figure 1:**
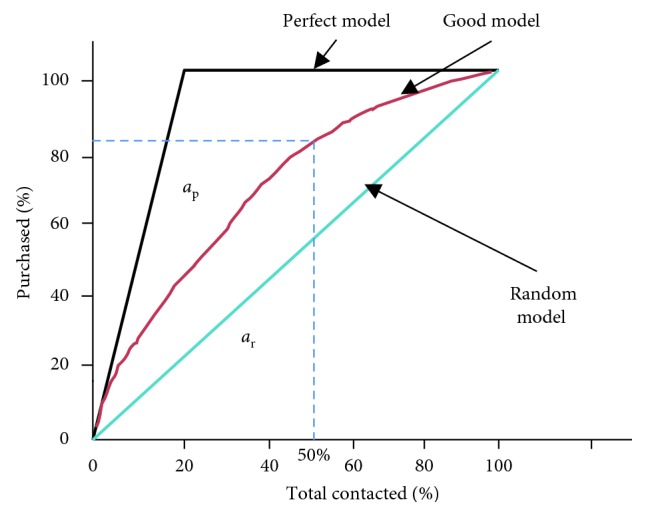
CAP curve analysis.

The CAP curve analysis is used for future forecasting of the data as well as to assess its predictive power [20]. Considering a scenario of 100K customers upon their likelihood to purchase a product which is presumably around 10%, a CAP curve analysis trains on known dataset and builds a model which can predict how much is a person likely to purchase the product given his previous behaviors or behaviors of customers similar to them. Now the company rather than taking random samples can segment these customers into categories and target only those who would increase their profits. A CAP curve analysis with a contrast between random and ideal sample is shown in Figure 1. According to the figure, the model gives accuracy of about 89% (after contacting 50% of the total customers, there is a probability that 89% of the total contacted customers will purchase the product). The more the curve is inclined towards the random line, the poorer is the model. The more the curve is inclined towards the ideal or perfect line, the better is the model.

The percentage area occupied by the CAP curve of the rating model is called the accuracy ratio (AR) [21]. It is calculated by dividing area under random curve to area under perfect curve. The rating of model according to intuitive X% is given in Table 4.

Overfitting in a model signifies that the data corresponds very closely or exactly towards the training set and therefore may fail to fit new data or predict the future in a reliable manner or the accuracy of the model in test dataset might decrease substantially.

## 3. Materials and Methods

### 3.1. Dataset

An experimental dataset consists of 10k customers of a bank for training and 1000 customers for testing to analyze their churn rate. The input predictors provided are given in Table 5.

The independent predictors were converted into dummy variables and transformed to fit the stepwise logistic regression model better and to avoid multicollinearity. For feature selection, the stepwise logistic regression model has been constructed in three ways: backward elimination model, forward selection model, and bidirectional model, and these models are contrasted based on their selection criterion, accuracy, and multicollinearity to choose the best model. Although the dataset contained very low correlation among its variables, the selection criteria showed increase in the case of the bidirectional model. Also, the accuracy drops from training to test data was lower in the bidirectional selection approach than the other two approaches due to which the former model is selected for further prediction analysis.

### 3.2. Flowchart of the Proposed Framework

**Figure 2:**
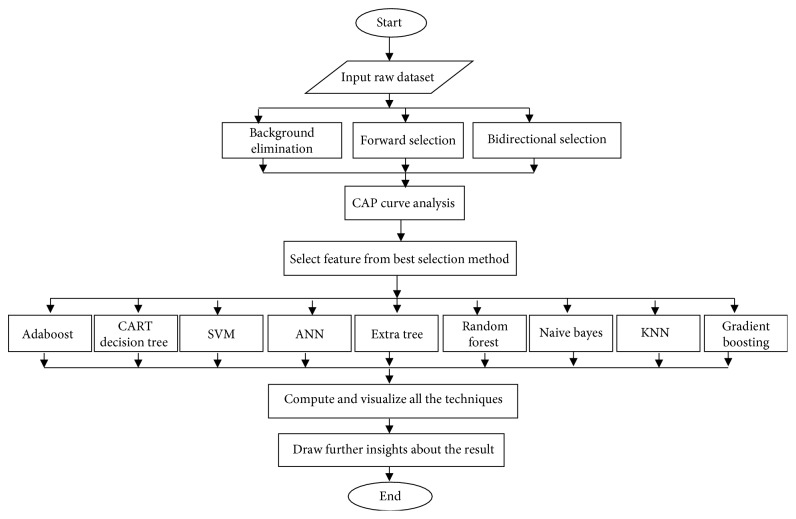
Flowchart of the proposed framework.

**Figure 3:**
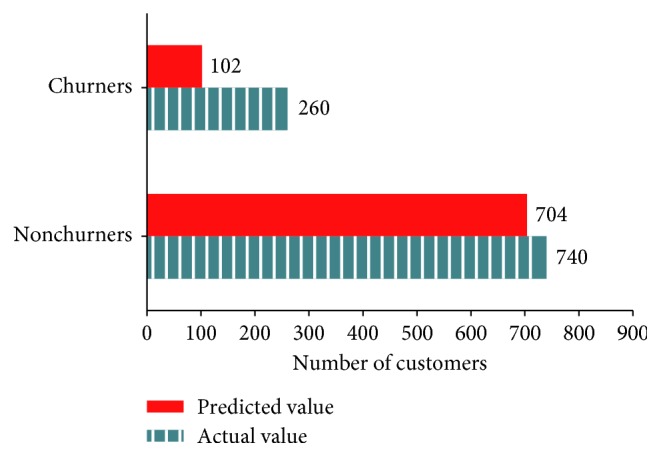
Classification of actual and predicted churners by AdaBoost.

**Figure 4:**
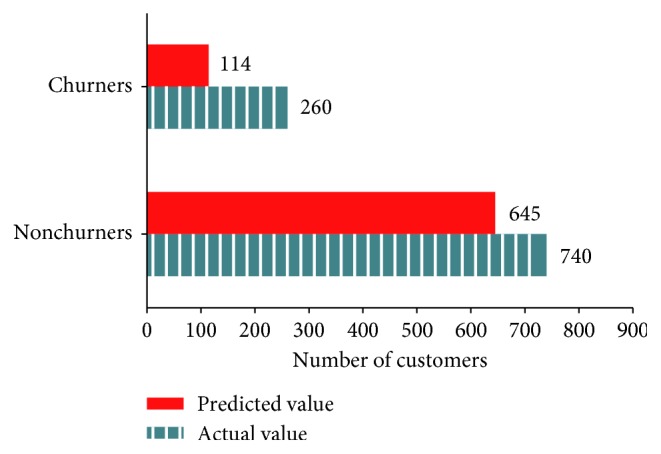
Classification of actual and predicted churners by the CART decision tree.

**Figure 5:**
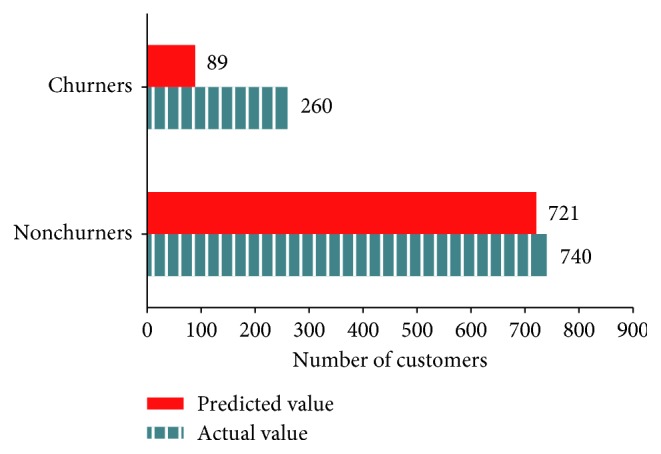
Classification of actual and predicted churners by SVM.

**Figure 6:**
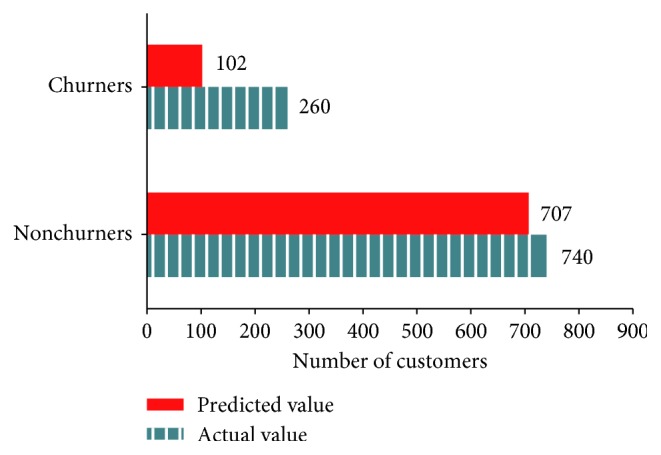
Classification of actual and predicted churners by ANN.

**Figure 7:**
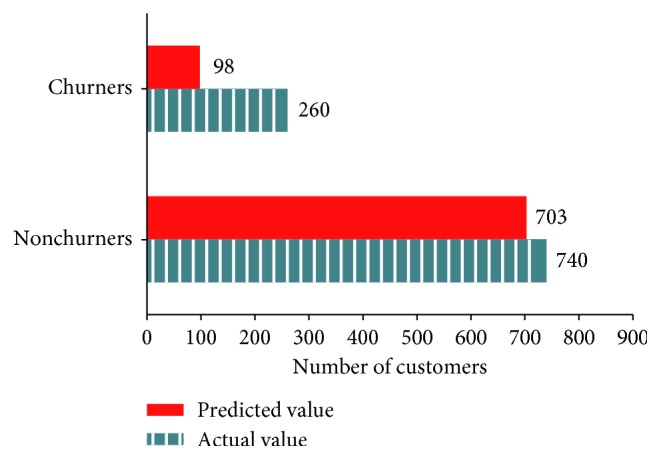
Classification of actual and predicted churners by extra trees.

**Figure 8:**
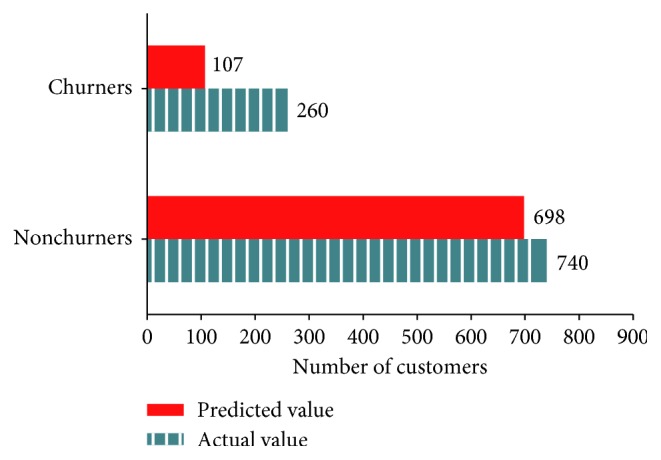
Classification of actual and predicted churners by random forest.

**Figure 9:**
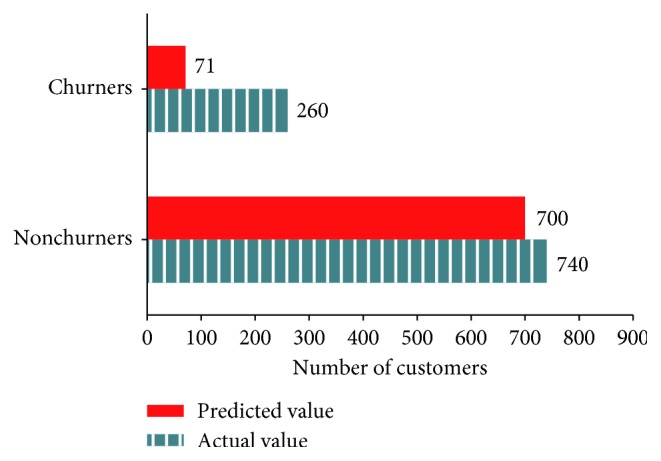
Classification of actual and predicted churners by Naïve Bayes.

**Figure 10:**
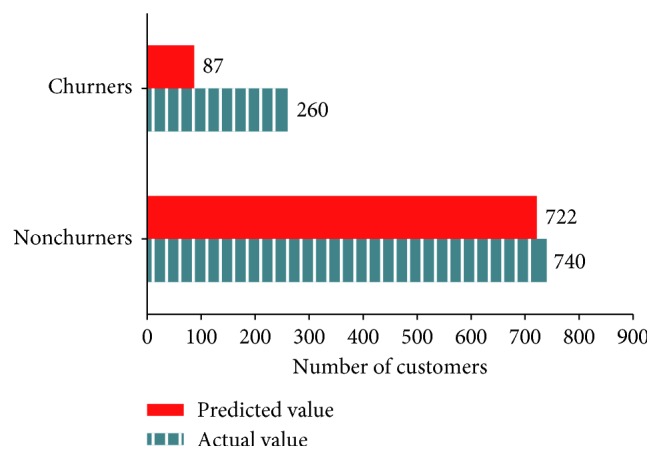
Classification of actual and predicted churners by KNN.

**Figure 11:**
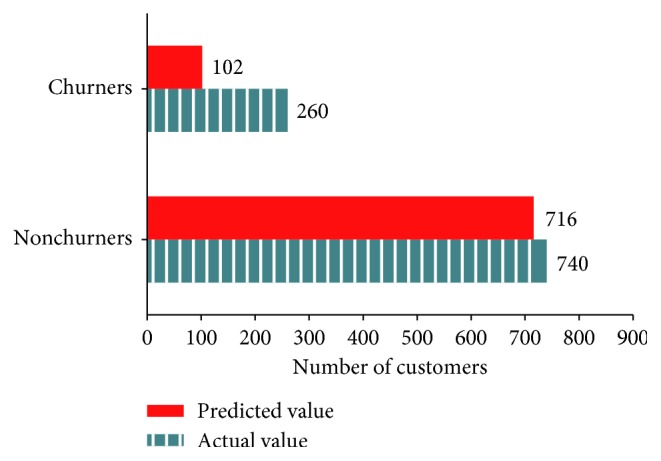
Classification of actual and predicted churners by gradient boost.

Figure 2 shows the flowchart of the proposed framework. It gives an outline of the whole paper. The dataset is simply the bank customer dataset that was introduced in the previous subsection. It is passed through the three feature selection approaches including background elimination, forward selection, and bidirectional selection. Next, their results are compared in terms of their accuracy to find the best approach. The features selected by the best approach are passed onto the next phase. Then, the nine different classification algorithms including Adaboost, CART decision tree, SVM, ANN, extra tree, random forest, Naïve Bayes, KNN, and gradient boosting are trained on this selected feature data of 10,000 rows, and their accuracy is tested using the test data which has 1,000 rows. Each technique is visualized using the graphs given in Figures 3–11. Also, the confusion matrix for is built each technique. Finally, some insights are discussed in Section 5 about the results achieved in the previous stages.

### 3.3. Impact of Dummy Variables and Transformation of Predictors

Each categorical predictor in the experimental dataset, i.e., gender and geography were converted into dummy variables which act as switches. For one predictor having *k* categorical values, *k*−1 dummy variables were produced and added to the model as the variable left will become the default situation for this regression model and its coefficient will be included in the constant. Including all the dummy variables in the equation would cause dummy variable trap because that would mean duplicating the variable and it would cause multicollinearity. This would cause the model to fail.

Some predictors were transformed to make them better fit for the model, to make their effects on the model more consistent, and to avoid correlation among the predictors.  Table 6 shows dummy variables and transformed variables.

### 3.4. Impact of Regression Approach

Table 3 shows the contrast between three constructed models of logistic stepwise regression based on selection of variables, correlations among their variables, goodness of fit criteria, and accuracy according to confusion matrix. CAP curve analysis on training and test data revealed no overfitting of the variables and the least accuracy drop was found in bidirectional model due to which it is the best model for feature selection for the experimental dataset.

### 3.5. Impact of Selection Criterion

Equation (1) shows the working of adjusted *R*^2^ approach. *R*^2^ never decreases despite whether the newly added variable inflates the model or deflates it. Therefore, it is biased and can never guarantee the significance of predictors. To cope with this problem, adjusted *R*^2^ was deduced:

(1) $$\begin{array}{c} \text{adjusted}\;\;R^{2}=1-\left[\frac{\left(1-R^{2}\right)\times \left(N-1\right)}{\left(N-p-1\right)}\right], \end{array}$$

where *R*^2^ = sample *R* [18], *p* is the number of predictors, and *N* is the total sample size.

Adjusted *R*^2^ has a penalizing factor. It penalizes for using independent variables that has no correlation with the target factor. So, if the factor is not helping the model, *p* will increase but comparatively *R* will have an insignificant increase and the adjusted *R*^2^ will decrease. On the other hand, if a factor is helping the model, *p* will increase but *R*^2^ will increase substantially overcoming *p*. So, the overall adjusted *R*^2^ will increase indicating that the model is good. So, adjusted *R*^2^ runs a fair battle over *R*^2^ which always increases and therefore is biased.

## 4. Observations and Results

### 4.1. Feature Selection Experiment

The consumer data can contain a huge number of predictors or input variables that might not all be significant in analyzing the churn rate. Regression techniques can help analysts or market researchers to eliminate irrelevant variables and figure out the best set to be used for building predictive models. Once the regression equation is formulated, its predictive ability can be examined using goodness of the fit criterion. The feature selection process via stepwise logistic regression minimizes dimensionality and effectively reduces costs and data volume. Identifying customer churn determinants, such as core service failures, customer complaints, loyalty programs, and service usage may help managers of the company improve the operations. A sophisticated and mature industry would opt to score every customer with the probability of churn and address the top *N* ones. CAP curve analysis helps in determining these top *N* customers who are at high level of leaving. It is also used to handle model deterioration over time, determining accuracy and check for overfitting of the data. Prediction analysis is one of the most powerful tools provided to data analytics by machine learning algorithms to construct a model that can predict the customer churn rate beforehand. Many classification techniques exist for this kind of supervised learning such as SVM, NN, decision trees, and gradient boosting, and based on their execution speed and their accuracy, each model can select the technique that fits best to its data.

### 4.2. Performance Experiment

Table 7 provides the predictive accuracy of the geodemographic segmentation models by different classification techniques.

### 4.3. Visualization Graphs

#### 4.3.1. AdaBoost

Figure 3 presents the number of churners and nonchurners predicted by AdaBoost and the actual number of those consumers in the testing dataset (predicted value = 806, actual value = 1000, and accuracy = 80.6%)

#### 4.3.2. CART Decision Tree

Figure 4 presents the number of churners and nonchurners predicted by the CART decision tree and the actual number of those consumers in the test data (predicted value = 759, actual value = 1000, and accuracy = 75.9%).

#### 4.3.3. Support Vector Machine (SVM)

Figure 5 presents the number of churners and nonchurners predicted by SVM and the actual number of those consumers in the test data (predicted value = 810, actual value = 1000, and accuracy = 81.0%).

#### 4.3.4. Artificial Neural Network (ANN)

Figure 6 presents the number of churners and nonchurners predicted by ANN and the actual number of those consumers in the test data (predicted value = 809, actual value = 1000, and accuracy = 80.9%).

#### 4.3.5. Extra Trees

Figure 7 presents the number of churners and nonchurners predicted by extra trees and the actual number of those consumers in the test data (predicted value = 801, actual value = 1000, and accuracy = 80.1%).

#### 4.3.6. Random Forest

Figure 8 presents the number of churners and nonchurners predicted by random forest and the actual number of those consumers in the test data (predicted value = 805, actual value = 1000, and accuracy = 80.5%).

#### 4.3.7. Naïve Bayes

Figure 9 presents the number of churners and nonchurners predicted by Naïve Bayes algorithm and the actual number of those consumers in the test data (predicted value = 771, actual value = 1000, and accuracy = 77.1%).

#### 4.3.8. KNN

Figure 10 presents the number of churners and nonchurners predicted by KNN and the actual number of those consumers in the test data (predicted value = 809, actual value = 1000, and accuracy = 80.9%).

#### 4.3.9. Gradient Boost

Figure 11 presents the number of churners and nonchurners predicted by KNN and the actual number of those consumers in the test data (predicted value = 818, actual value = 1000, and accuracy = 81.8%).

### 4.4. Model Predictions

Table 8 provides the confusion matrix of the geodemographic segmentation models by different classification techniques.

### 4.5. Effect of Feature Selection on Execution Time

Table 9 provides the difference in execution time of different classification techniques before and after feature selection process.

## 5. Discussion

### 5.1. Feature Selection

The bidirectional stepwise regression approach was chosen as the feature selection technique based on goodness of fit criterion (adjusted *R*^2^) and its accuracy and the intuition of features selected. Also, bidirectional model does not have suppressor affects unlike the others.

### 5.2. Multicollinearity

Based on variance inflation factors (VIF) and the CAP curve analysis conducted on training and test data, the data show no multicollinearity among predictors.

### 5.3. Future Forecasting

The CAP curve analysis resulted in 80.5% accuracy on the training set and 78.5% on the test set at 50% of the people contacted. This means that from the probable number of churners, the model will correctly predict 80% of the people likely to leave the bank when only contacted 50% of the total consumers. This will help the bank to manage marketing costs and gain higher profits.

Feature selection processes have an impact on decreasing the execution time of the classification algorithms used in the prediction analysis as per Table 9. Based on observations and analysis conducted through various classification techniques, gradient boosting turned to be the most promising approach according to the dataset.

**Figure 12:**
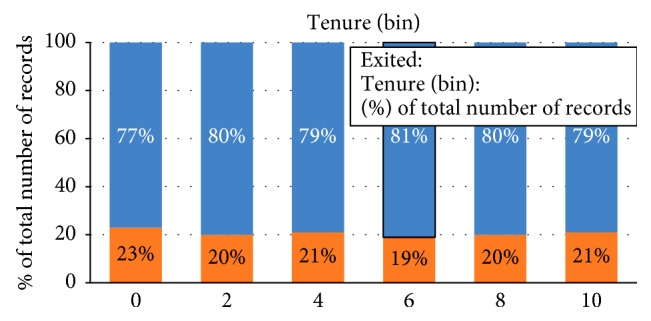
Classification of customers according to their tenure.

Tenure should have been a significant variable, but it is not as given by Tableau. But still, it was kept in the model because the adjusted *R*^2^ went down after removing it. This means that although tenure is contributing towards the fitness of the model, it is still insignificant. This means that the bank's policies might not favor or align with the loyalty of the consumers due to which customers might have been leaving (Figure 12).

**Figure 13:**
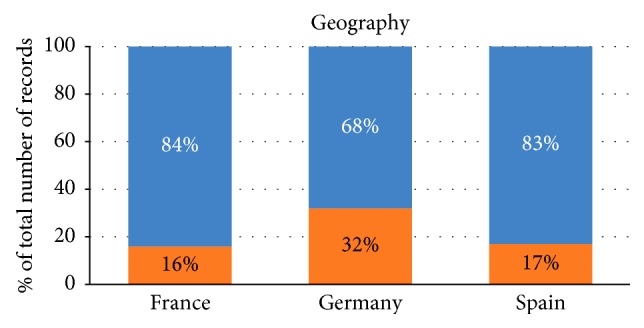
Classification of customers according to their geographic location.

People in Spain and France are equally likely to leave but people in Germany have a high probability of leaving the bank as shown by the magnitude of significant variables (Figure 13) by using the customer to business (C2B) and business to customer (B2C) model based on the geographical gaps in the market, distribution channels, geographical preferences, and geographical potential of the market.

## 6. Conclusions

In this paper, a simple geodemographic segmentation model was constructed using three methods of stepwise logistic regression, namely, backward elimination model, forward selection model, and bidirectional selection model to select the best model in accordance to their feature selection, accuracy, and confusion matrix. The predictive ability of the models is computed by CAP curve analysis, and insights have been drawn from that analysis. The next stage included prediction using various techniques like SVM, NN, and decision trees and clustering the churners on their likelihood of churn to strategies their retention. There is still extensive work to do from both a technique and business point of view. To further improve performance, other classification methods as well as other techniques addressing class rarity should be used and compared; for example, will the performance be improved by a hybrid of different classifiers or by building a multiple boosted regression model using the sampling technique. Accurate churn prediction only provides a basis for generating lists and prioritizing contact customers. So, the next stage of this research can involve performing a deeper analysis into the customer data to try to establish a new churn prediction retention model that will use the predicted and clustered data to assign a suitable retention strategy for each churner type. Identifying the reason for a particular customer's churn behavior and providing what the customer really needs are also important for targeted marketing research. The inclusion of additional input variables such as extracting service logs and customer complaints into our proposed technique might further enhance its predictive effectiveness. Constant relearning or rediscovery of a churn prediction model is required due to the evolving nature of the customers. The provision of a consumer-centric data warehouse would be desirable supporting the described knowledge maintenance requirement. In addition, churning is not restricted only to the bank industry but is also a great concern for other industries like telecommunications and Internet service providers, where stiff competition provides incentives for customers to switch. Thus, expanding the developed technique to other industries suggests interesting directions for future research.

## Figures and Tables

**Table 1:** A confusion matrix.

	Predicted: no	Predicted: yes
Actual: no	TN = 7687	FP = 276
Actual: yes	FN = 1597	TP = 440

**Table 2:** A correlation matrix.

Log_WA	Wealth accumulation	Log_Balance	Age
1.0000	0.8889	0.9984	−0.0075
—	1.0000	0.8651	−0.2463
—	—	1.0000	0.0345
—	—	—	1.0000

**Table 3:** Comparison among models for feature selection.

Features	Backward elimination		Forward selection		Bidirectional selection	
Features selected	CreditScore Age NumOfProducts IsActiveMember Female Germany Tenure Log_Balance		CreditScore Age NumOfProducts IsActiveMember Female Germany Log_Balance		CreditScore Age NumOfProducts IsActiveMember Female Germany Tenure Log_Balance	
Adjusted *R*-squared	0.151006		0.149718		0.152092	
Correctly predicted cases	8127 (81.3%)		8122 (81.1%)		8127 (81.3%)	
CAP curve (training data)	Accuracy, 81%		Accuracy, 80.5%		Accuracy, 80.5%	
CAP curve (test data)	Accuracy, 78%		Accuracy, 77%		Accuracy, 78%	
Accuracy drop	3%		3.5%		2.5%	
Multicollinearity	CreditScore	1.001	CreditScore	1.001	CreditScore	1.001
	Age	1.012	Age	1.012	Age	1.012
	NumOfProducts	1.151	NumOfProducts	1.151	NumOfProducts	1.151
	IsActiveMember	1.010	IsActiveMember	1.009	IsActiveMember	1.010
	Female	1.003	Female	1.003	Female	1.003
	Germany	1.269	Germany	1.269	Germany	1.269
	Tenure	1.001	Log_Balance	1.420	Tenure	1.001
	Log_Balance	1.420			Log_Balance	1.420

**Table 4:** Rating of model according to the accuracy ratio.

AR (%)	Rating
90–100	May be overfitted
80–90	Very good
70–80	Good
60–70	Poor
Less than 60%	Very poor

**Table 5:** Input variables.

Id	Variable name	Description
1	Row number	Row number
2	CustomerId	ID of the customer
3	Surname	Surname of the customer
4	CreditScore	Credit score of the customer
5	Geography	Region where the customer is located
6	Gender	Gender of the customer
7	Age	Age of customer
8	Tenure	Number of years the customer has been associated with the bank
9	Balance	Balance in the customer's account
10	NumOfProducts	Number of products
11	HasCrCard	True, if the customer has credit card and vice versa
12	IsActiveMember	True, if the customer is an active member and vice versa
13	Estimated salary	Estimated salary of the customer
14	Exited	True, if the customer has exited and vice versa

**Table 6:** Dummy variables and transformed variables.

ID	Variable name	Description
5	Germany	Dummy variable-Germany
6	France	Dummy variable-France
6	Female	Dummy variable
9	LogBalance	Log 10 (balance + 1)

**Table 7:** Accuracy and error rates of different classification techniques.

Technique	Accuracy	Error rate
CART decision tree	75.9% (759/1000)	24.1% (241/1000)
Random tree	80.5% (805/1000)	19.5% (195/1000)
Gradient boost	81.8% (818/1000)	18.2% (182/1000)
AdaBoost	80.6% (806/1000)	19.4% (194/1000)
Extra tree	80.1% (801/1000)	19.9% (199/1000)
SVM	81.0% (810/1000)	19.0% (190/1000)
ANN	80.9% (809/1000)	19.1% (191/1000)
Naïve Bayes	77.1% (771/1000)	22.9% (229/1000)
kNN	80.9% (809/1000)	19.1 (191/1000)

**Table 8:** Confusion matrix of different classification techniques.

Model	Actual class	Actual prediction	
		Nonchurners	Churners
CART decision tree	Nonchurners	645/740 (87.16%)	95/740 (12.84%)
	Churners	146/260 (56.15%)	114/260 (43.85%)
Random forest	Nonchurners	698/740 (94.32%)	42/740 (5.68%)
	Churners	153/260 (58.8%)	107/260 (41.15%)
Gradient boost	Nonchurners	716/740 (96.76%)	24/740 (3.24%)
	Churners	158/260 (60.77%)	102/260 (39.23%)
AdaBoost	Nonchurners	704/740 (95.14%)	36/740 (4.86%)
	Churners	158/260 (60.77%)	102/260 (39.23%)
Extra trees	Nonchurners	703/740 (95.0%)	37/740 (5.0%)
	Churners	162/260 (62.31%)	98/260 (37.69%)
SVM	Nonchurners	721/740 (97.43%)	19/740 (2.57%)
	Churners	171/260 (65.77%)	89/260 (34.23%)
Artificial neural network	Nonchurners	707/740 (95.54%)	33/740 (4.46%)
	Churners	158/260 (60.77%)	102/260 (39.23%)
Naïve Bayes	Nonchurners	700/740 (94.59%)	40/740 (5.41%)
	Churners	189/260 (72.69%)	71/260 (27.31%)
kNN	Nonchurners	722/740 (97.56%)	18/740 (2.44%)
	Churners	173/260 (66.54%)	87/260 (33.46%)

**Table 9:** Effect of feature selection on execution time.

Technique	Full dataset (sec)	Feature selected data (sec)	Decrease (%)
CART decision tree	0.071	0.044	37.392
Random forest	1.728	1.344	22.189
Gradient boost	0.921	0.673	26.943
AdaBoost	1.103	0.870	25.155
Extra trees	1.175	0.990	15.758
SVM	3.458	3.120	9.771
Artificial neural network	458.321	439.653	4.073
Naïve Bayes	0.004	0.002	31.906
kNN	0.012	0.012	4.460

## Data Availability

The data used to support the findings of this study are available from the corresponding author upon request.
